# Replicated evolutionary inhibition of a complex ancestral behaviour in an adaptive radiation

**DOI:** 10.1098/rsbl.2018.0647

**Published:** 2019-01-30

**Authors:** Susan A. Foster, Shannon O'Neil, Richard W. King, John A. Baker

**Affiliations:** 1 Department of Biology, Clark University, Worcester, MA 01610, USA; 2 Division of Planning and Permitting, Frederick County, Frederick, MD 21701, USA

**Keywords:** diversionary display, *Gasterosteus aculeatus*, phenotypic plasticity, threespine stickleback

## Abstract

Adaptive radiations often exhibit high levels of phenotypic replication, a phenomenon that can be explained by selection on standing variation in repeatedly divergent environments or by the influence of ancestral plasticity on selection in divergent environments. Here, we offer the first evidence that plastic loss of expression of a complex display in a novel environment, followed by selection against expression, could lead to replicated evolutionary inhibition of the phenotype. In both ancestral (oceanic) and benthic (freshwater) populations of the threespine stickleback fish, cannibalism is common and males defending nests respond to approaching groups with a complex diversionary display. This display is not exhibited by males in allopatric, limnetic (freshwater) populations from which cannibalistic groups are absent. Laboratory-reared males from three limnetic populations exhibit a reduced tendency to respond to cannibalistic foraging groups relative to laboratory-reared ancestral and benthic males, but still are capable of producing a similar array of forms of the display despite many generations of disuse. Thus, replication in adaptive radiations can reflect reduced expression of an ancestral trait followed by evolutionary inhibition while the population retains the capacity to express the trait under extreme ancestral conditions.

## 1. Introduction

High levels of replication in adaptive radiations have historically been considered the products of direct selection on standing genetic variation that increases the match between common environments and the populations or species that invade them. This has typically been viewed as a constructive process in the sense that ancestral phenotypes are modified to match their environments adaptively and differentially [1,2]. An alternative possibility not often discussed is that replicated phenotypes can be a consequence of the loss of expression of ancestral phenotypes in a particular environmental context. Thus, ancestral phenotypes could consistently decay in particular environments, leading to phenotypic replication. This could involve a complete loss of the capacity to express the phenotype, or in the case of phenotypically plastic traits, the ability to express the phenotype could be retained, but the stimulus needed to elicit it may have disappeared from the environment, causing an apparent loss of capacity to express the phenotype although that capacity is retained [1–4]. If a plastic phenotype is maladaptive in the novel environment when expressed, selection could favour evolutionary inhibition of expression of the trait, increasing the difficulty of understanding the processes that have led to replicated evolution in adaptive radiations.

Assessing directionality (trait acquisition or loss) in the evolution of replicated phenotypes in adaptive radiations is difficult because traits must be evaluated in an ancestor and in multiple, derived populations in at least two ecotypes demonstrating replication [4–7]. When traits exhibit plasticity, particularly short-term, rapid activational plasticity (*sensu* [8]), rather than constitutive expression, the problem is exacerbated as the ancestor or a reasonable live proxy for the ancestor must be available for study [4,7,9]. Here, we take advantage of the adaptive radiation of threespine stickleback, *Gasterosteus aculeatus.* Following the last glacial retreat, oceanic stickleback colonized freshwater habitats, repeatedly and independently, giving rise to similar populations in similar habitats as exemplified by the well-studied benthic–limnetic axis [5,10,11]. Benthic populations retain the ancestral (oceanic) tendency to form benthic foraging groups on breeding grounds. They are attracted to the nests by nesting and courtship activity, and attack nests guarded by males, destroying the nests and cannibalizing embryos or fry in the nests if present [6,12]. Males perform complex, conspicuous diversionary displays at the approach of groups in oceanic and benthic populations that, if effective, draw the groups from the nest [12]. Extreme limnetic populations are planktivorous and non-cannibalistic, and limnetic males court females within groups, rather than performing diversionary displays. Although our early research indicated evolutionary loss of the diversionary display [6,12], we here examine the possibility that at least some limnetic males retain the capacity to perform diversionary displays despite generations of disuse.

## 2. Material and methods

In May 2009 and 2010, wild-caught adult stickleback from nine populations (table 1) were used to create multiple families from each population, each with a different pair of parents. Embryos were shipped to Clark University and reared for 1–2 years. Embryo rearing procedures followed standard laboratory protocols [13]. Transition from short to long day length in June 2011 brought adult males into reproductive condition. As they assumed nuptial coloration, signalling reproductive readiness, males were placed in 38-l aquaria with a nesting dish at one end and material for nest building. Once the nest was built, a transparent 12.5-cm-diameter cylinder covered in a black plastic sleeve was placed at the opposite end of the aquarium from the nest. Five foragers were placed in the cylinder and allowed to acclimate for 10 min during which male activity was videotaped from the long face of the aquarium. The sleeve was removed and bloodworms were released over the nest. When at least one individual in the cylinder had oriented towards the nest, the cylinder was removed and interactions between the group, which typically attacked the nest immediately (electronic supplementary material, videos), and the male were videotaped for 5 min. When possible, up to three males were tested from each family, one with each foraging group. All video tapes were scored blind by S.O. to ensure uniformity of scoring and eliminate bias. Although all five diversionary display types [12] were recorded, only three, the upright swim root (USR), erratic swim root (ESR) and side swim root (SSR) were common enough to be included in analyses (electronic supplementary material, videos).

**Table 1.** Names, acronyms, and locations of the nine study populations in Alaska (AK) and British Columbia (BC). The number of males from each population tested with each of the three foraging group populations is given as N1–N2–N3 (Crystal Lake, Kalmbach Lake and Willow Lake foragers, respectively).

ecotype	population	latitude, longitude	foraging group (N's)
benthic	Beverly Lake (AK1)	61.615 N −149.574 W	6–7–4 (17)
	Stepan Lake (AK2)	61.571 N −149.815 W	9–9–7 (25)
	Willow Lake (AK3)	61.745 N −150.055 W	6–10–5 (21)
	Crystal Lake (BC1)	49.044 N −123.957 W	9–9–7 (25)
ancestral	Resurrection Bay (AK4)	60.123 N −149.414 W	8–7–8 (23)
	Rabbit Slough (AK5)	61.536 N −149.253 W	5–5–5 (15)
limnetic	Lynne Lake (AK6)	61.711 N −150.046 W	6–2–3 (11)
	Garden Bay Lake (BC2)	49.647 N −124.015 W	11–11–7 (29)
	North Lake (BC3)	49.750 N 123.971 W	8–9–6 (23)

Foraging groups comprised five individuals randomly selected from one of three large holding tanks each containing mixed family assemblages of adults from one of three benthic populations (table 1). Each group of five was tested only once per day, and all were returned to their population holding tanks at the end of the day for reuse in ensuing trials. When three males could be tested from a family, one male was exposed to each of the three foraging populations. Details are given in table 1.

The probability that a male would perform at least one diversionary display in response to an attack was analysed via GLMM, using a logit link function applied to the binomial response set (yes, no). Ecotype and foraging group identity, and their interaction, were fixed factors in the analysis, with population random. Owing to the low and unequal number of families available per population per foraging group, a family term was not included. Males often made more than one diversionary display per trial, and differences in frequencies were similarly probed using a GLMM assuming a Poisson distribution of counts, with ecotype and foraging population fixed and population random. Differences in the frequency of the three most common diversionary display types were tested within a two-way GLMM, with ecotype and diversionary type as fixed factors and population random, assuming a Poisson distribution of counts.

## 3. Results

**Figure 1.**
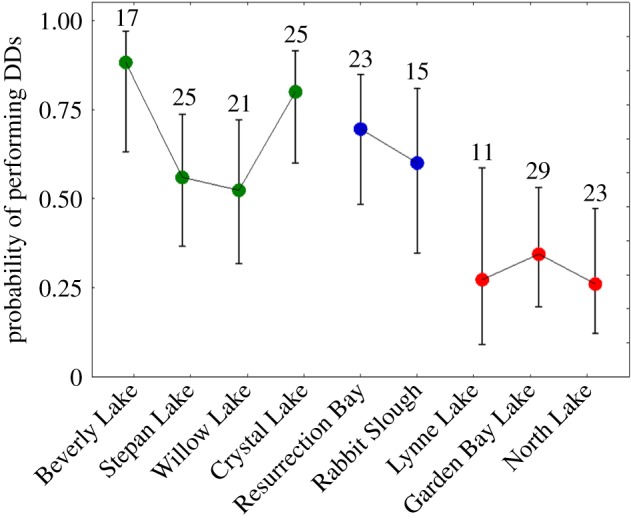
Predicted probability of performing at least one diversionary display (DD) for males from nine populations of stickleback (with 95% confidence intervals). Limnetic males (red) were significantly less likely to display than were oceanic (blue) or benthic (green) males, which did not differ. The number of males tested is indicated over each mean. Population geographical locations are given in table 1.

**Figure 2.**
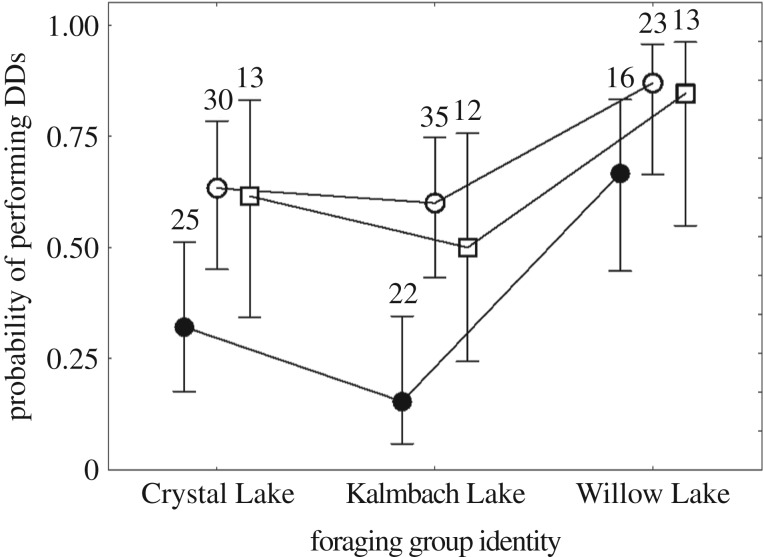
Males from the three ecotypes responded to foraging groups from Willow Lake more strongly than they did to those from the other two lakes. Filled circles indicate limnetic males; open circles indicate benthic males; open squares indicate oceanic males; whiskers indicate 95% confidence bounds. The number of males tested is given over each mean; all populations are pooled for each ecotype (table 1).

Some males within each of the populations responded to the foraging groups with diversionary displays, though predicted probabilities differed considerably across ecotypes (figure 1: *X*^2^ = 24.05, d.f. = 2, *p* < 0.0001). Responses to the foraging groups also differed (figure 2: *X*^2^ = 24.32, d.f. = 2, *p* < 0.0001), with Willow Lake foragers eliciting stronger responses than the other two populations. Benthic and ancestral ecotypes (not different; *p* > 0.05) were more likely to perform diversionary displays than limnetic males (*p* < 0.01), an observation that holds for each of the foraging group populations. Within-ecotype variation was also suggested (figure 1; *X*^2^ = 11.24, d.f. = 6, *p* = 0.08), with benthic populations contributing most to this added variance; the probability of benthic populations performing a diversionary display varied from well above 80% to just above 50%. Predicted probabilities for the three limnetic populations and two ancestral populations showed less variability. The mean number of the three major types of diversionary displays per trial differed across ecotypes (electronic supplementary material, figure S1: *X*^2^ = 74.9, d.f. = 2, *p* < 0.0001), but the three ecotypes used the three forms of the display with similar relative frequencies.

## 4. Discussion

Although male stickleback in allopatric limnetic populations do not perform diversionary displays at the approach of groups of conspecifics in nature, at least some in each population studied here are capable of performing these displays when their nest is attacked by foraging groups, as our data demonstrate. The increase in the display probability of limnetics when attacked by the Willow Lake groups compared with attacks by other groups parallels, but is greater in magnitude, than the responses of males from other ecotypes. This suggests that there may be a stimulus strength effect such that more aggressive groups are more likely to elicit displays, particularly from limnetics. As cannibalistic groups in nature are typically much larger than those we used here [12,14–16], larger groups foraging on benthos could also be more effective in eliciting displays than were the small, but aggressive groups used here.

Given the increase in responsiveness of limnetics to more closely approach that of the benthic and oceanic fish when confronted with the more aggressive Willow Lake groups, we suggest that the most probable explanation for the replicated ‘loss’ of the diversionary display in nature is consistent evolutionary inhibition of the ancestral diversionary display in limnetic populations. As all of our freshwater populations are in different watersheds, inhibition of expression must have evolved independently in each limnetic population (replication). This said, we cannot rule out the possibility that some limnetic individuals have lost the capacity to display entirely.

The apparent cause for the initial loss of expression in evolving limnetic populations is the retention of neotenic plankton feeding upon invasion of large oligotrophic lakes and the associated disappearance of ancestral cannibalism in these populations. This inference is supported by the continued appearance of diversionary displays in limnetic members of the stickleback species pairs, where these males experience offspring cannibalism by foraging groups of the benthic species [14]. Under these circumstances, retention of diversionary displays as defences against benthic foraging groups is expected as the threat persists, albeit from individuals of the benthic species. By contrast, evolutionary inhibition in allopatric limnetics likely stems from lost mating opportunities when males perform diversionary displays at the approach of groups rather than courting females within them, and potentially from an enhanced risk of predation as piscine predators are present in all populations ([13]; electronic supplementary material, natural history).

As in avian diversionary displays [15], those performed by stickleback incorporate elements co-opted from other aspects of behaviour [16]. For example, rooting (a foraging behaviour) is commonly performed at the end of displays. In more extreme forms of the display, motor patterns like those involved in nest gluing are incorporated, and in SSR displays that occur along the substratum, males tap the substratum with their snout as if displaying the nest entrance to a female. Again, as in avian diversionary displays [15], the original behaviours have become elaborated and ritualized, but the behavioural origins of the motor patterns are still apparent [12]. A probable explanation for the persistence of this disused behavioural display is that unexpressed behavioural traits, unlike constitutive armour traits, are buffered from selection [4]. Common genomic and neural circuits involved in co-opted components of the behaviour could inhibit evolutionary loss of the capacity to perform diversionary displays because disruption of the display could lead to disruption of behavioural elements co-opted from other behavioural contexts. This system thus offers an exciting opportunity to explore the possibility that constraints imposed by common genomic and neural networks tend to maintain ancestral behavioural structure, and may be unexpressed for long periods after which function reappears. Elegant recent examples involve the production of super soldiers in ants [17], and the persistence of neural substrates for walking that were present in cartilaginous fishes well prior to the invasion of land by bony fishes [18]. What is novel about the research presented here is that we provide evidence that inhibition or loss of ancestral phenotypes can lead to replication in adaptive radiations — directionality for this phenomenon that is rarely considered — and it opens the possibility of understanding the relative contributions of plasticity and selection on standing variation to this phenomenon.

## Supplementary Material

Natural history, figure and map

## Supplementary Material

Two foraging videos

## Supplementary Material

Two foraging videos

## Supplementary Material

Nine Diversionary display videos

## Supplementary Material

Nine Diversionary display videos

## Supplementary Material

Nine Diversionary display videos

## Supplementary Material

Nine Diversionary display videos

## Supplementary Material

Nine Diversionary display videos

## Supplementary Material

Nine Diversionary display videos

## Supplementary Material

Nine Diversionary display videos

## Supplementary Material

Nine Diversionary display videos

## Supplementary Material

Nine Diversionary display videos

## Data accessibility

Data have been made available online from the Dryad Digital Repository at: http://dx.doi.org/10.5061/dryad.2538mj0 [19].
