# Supplementary material for: Replicated evolutionary inhibition of a complex ancestral behaviour in an adaptive radiation
Source: Biol Lett. 2019 Jan 30;15(1):20180647. doi: 10.1098/rsbl.2018.0647 (PMC6371902; doi:10.1098/rsbl.2018.0647)
Supplement: Natural history, figure and map [file rsbl20180647supp1.pdf]

**Supplemental Information**

The study populations are either from the Strait of Georgia region of British Columbia Canada (BC populations) or the Cook Inlet region of Alaska USA (AK population). All can be precisely located using the latitude/longitude information provided for each (see also Supplemental figure 2). Each of our study lakes in each region is in a separate watershed, and thus gene flow between them is not possible. Several of our study lakes have no stream connection to the ocean through which oceanic fish could migrate. In the others, in-water behavioral observations over several years, combined with intensive annual collections for preserved specimens (10's of thousands of specimens), have given no indication of the presence of oceanic fish, with one exception—in a single year out of 22 we collected two oceanic fish in Stepan Lake, one of our benthic populations.

Oceanic and benthic populations both are cannibalistic as described in the manuscript. Cannibalism is chronic during the breeding season in the benthic populations we have studied, but more variable in the oceanic populations as settlement density varies and sometimes does not allow formation of effective foraging groups. When foraging groups are common, females typically initiate courtship by positioning themselves upon the dorsal surface of the male. If the male is receptive he will raise his spines and press them into the belly of the female while meandering in circles (dorsal pricking). If no groups are detected, the male may lead the female to the nest, placing his snout in the nest entrance "showing" her the nest as he turns on his side. She may then steal eggs and back out, or enter the nest and spawn. Occasionally, even if she enters the nest, she will not spawn and will leave via the exit. In oceanic populations when foraging groups are absent, and in limnetic populations, males initiate courtship with the conspicuous zig-zag dance. If the female responds positively and follows him, he may lead her directly to the nest, showing her the entrance. The outcomes are as already described. Males of all ecotypes may interrupt courtship with fanning or in the case of zig-zagging males, with bouts of dorsal pricking. Citations describing the ecotypes are in the main text.

The diversionary displays scored were of three types. In the Upright Swim Root (USR) the male simply swims directly off of his nest and roots vigorously in the substratum. The Erratic Swim Root (ESR) is similar except the swimming is very erratic and more conspicuous to human observers. In the Side Swim Root (SSR) the male swims on his side either high in the water column, or near the substratum, tapping his snout to the bottom as if showing the entrance to the nest.

**Video Tapes**

We include 9 exemplar video tapes of the display types by ecotype in addition to two videos of the behavior of groups when released from the clear tube, demonstrating nest attacks. All population and group identifiers were masked for scoring.

**Supplemental Figure 1**

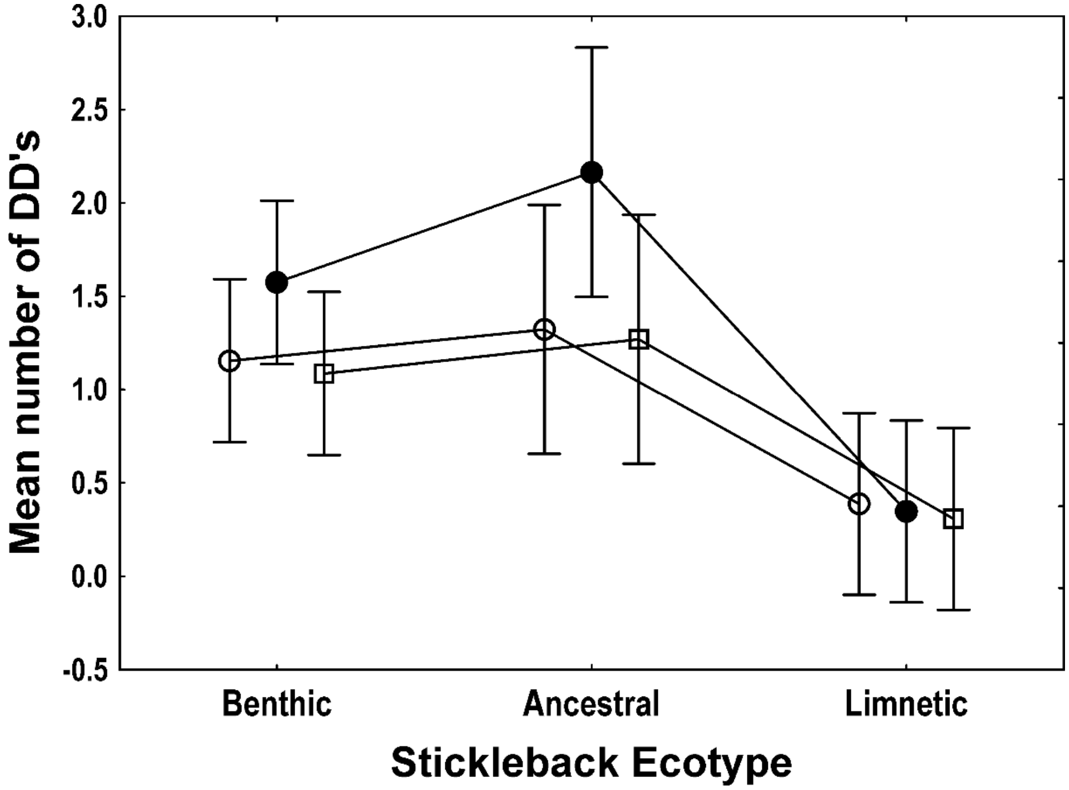

**Figure S1.** Although limnetic males performed significantly fewer diversionary displays overall (see main text), the three ecotypes performed similar proportions of each diversionary display type (GLMM Interaction test: Ecotype*Display Type, $X^2 = 3.66$, DF = 4, p = 0.45). Open circles (ESR); open squares (USR); filled circles (SSR); brief descriptions of each DD type are provided above.

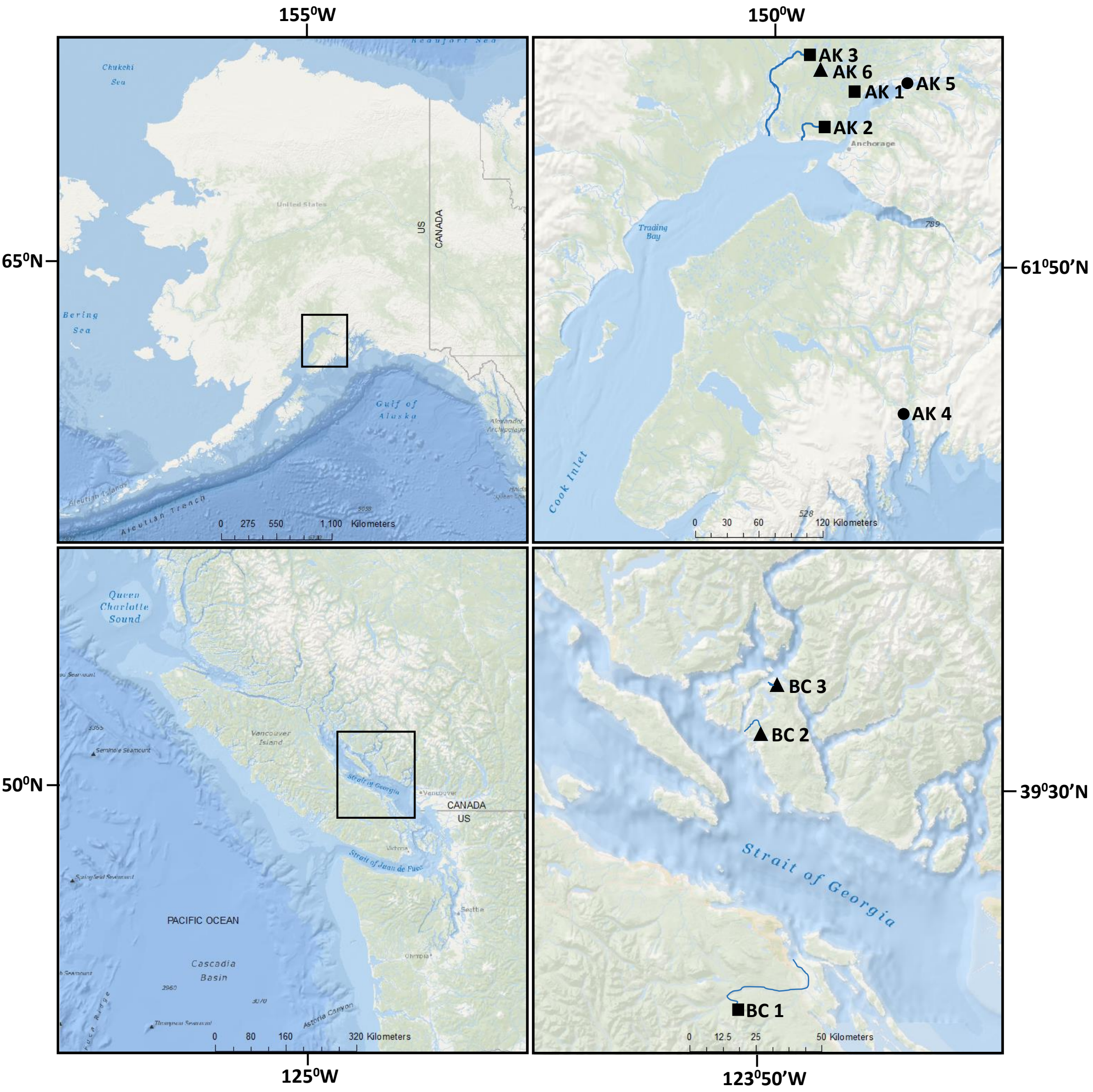


**Figure S2.** Panels show geographical context (left) and population locations (right) for Alaska (top) and British Columbia (bottom) study sites, including drainages. Two of the study lakes in Alaska lack outlet streams (AK1 & AK6), and ancestral (oceanic) populations are in tidal sloughs immediately adjacent to ocean inlets. Symbols represent benthic (squares), limnetic (triangles), or ancestral (circles) ecotypes and population acronyms are same as in text (Figure 1). All maps are displayed North up. Data Sources: ESRI, Garmin, GEBCO, NOAA, NGDC, National Geographic Society, HERE, Geonames.org, & other contributors. Maps created in ArcGIS (ESRI 2018 ArcGIS Desktop: Release 10.6.1 Redlands, CA., Environmental Systems Research Institute).
